# Surfing Across Life Domains: A Qualitative Study on the Role of Leisure Activities in Occupational Well-Being

**DOI:** 10.3390/ijerph23080979

**Published:** 2026-07-28

**Authors:** Cataldo Giuliano Gemmano, Mirko Pio Petruzzelli, Amelia Manuti

**Affiliations:** Department of Educational Sciences, Psychology, Communication, University of Bari “Aldo Moro”, 70121 Bari, Italy; mirkopetruzzelli28@gmail.com (M.P.P.); amelia.manuti@uniba.it (A.M.)

**Keywords:** nature-based activities, blue spaces, leisure activities, work-related stress, coping strategies, work–life interface, skill transfer, occupational well-being

## Abstract

**Highlights:**

**Public health relevance—How does this work relate to a public health issue?**

- Work-related stress and difficulties in managing the work–life interface represent key public health challenges affecting psychological well-being and long-term health outcomes.
- This study examines the role of extra-work contexts as sources of psychological recovery and stress regulation, extending traditional approaches to occupational health.

**Public health significance—Why is this work of significance to public health?**

- The findings highlight how nature-based leisure activities, such as surfing, may support coping and recovery processes among working adults.
- The study contributes to the growing evidence on nature-based activities as protective factors for mental health and well-being.

**Public health implications—What are the key implications or messages for practitioners, policy makers and/or researchers in public health?**

- Promoting access to nature-based and recovery-oriented activities may represent a complementary strategy for reducing work-related stress and supporting psychological well-being.
- Organizations and practitioners should consider employees’ extra-work experiences as part of integrated approaches to well-being and work–life management.

**Abstract:**

Work-related stress and difficulties in managing the work–life interface represent significant challenges for psychological well-being. While research has increasingly examined coping and recovery processes, less attention has been paid to the role of nature-based leisure activities in supporting these dynamics. This qualitative study explores how adult workers who regularly practice surfing use this activity to cope with work-related stress and how it relates to the development of personal resources and the work–life interface. Semi-structured interviews were conducted with 14 Italian workers, and the data were analyzed using thematic analysis. Three main themes emerged. First, surfing was described as a coping strategy that supports emotional regulation, psychological detachment from work, and recovery, particularly through immersion in a natural environment. Second, participants reported that experiences developed in surfing, such as managing uncertainty, risk, and decision making, were transferable to the work context. Third, surfing played a complex role in the work–life interface, contributing to balance but also generating time-based tensions when not adequately integrated into daily routines. Overall, the findings highlight the active role of individuals in using extra-work activities as resources for coping and recovery and underline the importance of considering nature-based experiences within broader models of occupational well-being and work–life dynamics.

## 1. Introduction

In contemporary workplaces, increasing job demands, accelerated work rhythms, and the diffusion of psychosocial risks have made employees’ well-being a central concern in occupational research [1,2]. Recent reviews confirm that employee health and well-being are shaped by a complex interplay of job demands, resources, recovery processes, and individual behaviors, rather than by workplace stressors alone [3]. In this context, a growing body of research has emphasized that well-being is not only shaped within the workplace, but also significantly influenced by experiences outside of work, particularly those that enable recovery from job-related stress [4,5]. This issue is particularly relevant in the Italian context, where psychosocial risk management is formally embedded in occupational safety and health practices, although recent evidence suggests that further attention is still needed to develop primary prevention interventions aimed at reducing job demands and work-related stress [6].

Extra-work activities, therefore, represent a critical domain for understanding how employees maintain and enhance their psychological well-being. Specifically, engaging in leisure activities during non-work time can contribute to workplace well-being through multiple mechanisms. These activities can facilitate recovery processes by allowing individuals to restore depleted cognitive and emotional resources, while also promoting positive affect and mood regulation that may buffer the impact of stress [7,8]. Moreover, extra-work experiences can play a key role in shaping the work–life interface by generating personal resources and influencing how individuals manage the relationship between work and non-work domains [4,9]. Through these mechanisms, leisure activities may not only support coping with work-related stress but also contribute to overall psychological well-being and functioning at work [10,11].

Among extra-work activities, increasing attention has been devoted to outdoor and nature-based experiences. Research in environmental psychology suggested that natural environments support psychophysiological recovery by promoting attentional restoration and stress reduction [12,13]. In particular, so-called blue spaces (i.e., environments characterized by the presence of water) have been associated with enhanced subjective well-being, reduced stress, and higher perceived restorativeness [14,15]. Recent systematic reviews further support the relevance of nature-based activities and interventions for mental health and well-being, while also emphasizing the need to better understand the conditions and mechanisms through which these experiences produce benefits [16,17]. Building on this evidence, the Blue Mind framework [18] proposes that interaction with water fosters states of calm, clarity, and connection, contributing to an embodied form of well-being in which cognitive, emotional, and physical processes are intertwined.

Within this landscape, surfing represents a particularly relevant, yet underexplored, form of extra-work activity. Surfing combines physical exertion, immersion in a dynamic natural environment, and sustained attentional focus on the present moment. Surfing was selected as the focal activity because it combines several features that are theoretically relevant for occupational well-being: immersion in a blue-space environment, physical effort, exposure to changing and uncertain conditions, and sustained attentional focus. Previous studies have described surfing as an experience associated with mindfulness-like states, active coping, and flow [19,20,21]. These characteristics suggest that surfing may represent a powerful resource for both recovery and enrichment processes across life domains.

However, despite growing interest in nature-based well-being and surf-related research, empirical studies have largely focused on clinical populations or structured surf therapy interventions [19,22], overlooking adult workers who engage in surfing as a regular leisure activity. As a result, little is known about how extra-work activities such as surfing may function as spontaneous, everyday resources for coping with work-related psychosocial risks, supporting recovery processes, and fostering work–life balance. This gap is relevant both theoretically, by limiting the integration between occupational health psychology and research on nature-based well-being, and practically, by overlooking accessible strategies for promoting psychological well-being in working populations.

To address this gap, the present qualitative study explores how adult workers who regularly practice surfing use this activity as a coping strategy to deal with work-related stress and enhance their psychological well-being. We analyze participants’ lived experiences through semi-structured interviews to understand how surfing, as a leisure activity, contributes to (a) coping with work-related stress, (b) the development of personal resources, and (c) the shaping of the work–life interface. Accordingly, the study was guided by the following research questions: How do adult workers experience surfing as a resource for coping with work-related stress? What personal resources do they perceive as being developed through surfing and transferred to the work domain? How does surfing shape the relationship between work and non-work life domains?

This research is grounded in an integrated perspective, combining coping theory and the work–life interface framework [9,23,24], which conceptualizes how individuals manage demands across work and non-work domains. Within this perspective, surfing is conceptualized as a leisure activity that may function as a resource for coping with work-related demands and facilitating recovery processes [4,23], while also shaping the work–life interface and supporting the development and transfer of personal resources across domains [11,24]. Specifically, the physical, emotional, and cognitive experiences associated with surfing may enhance psychological well-being and mitigate the impact of work-related psychosocial risks [19,20,21].

Consistent with this perspective, prior evidence showed that physical activity in natural environments, particularly in marine settings, was associated with improved mental health, reduced stress, and increased positive affect [14,15]. Studies on surf-related experiences also highlighted improvements in self-esteem, emotional regulation, and stress reduction [19,22], suggesting that surfing might foster a form of embodied mindfulness, that is, a state of present-focused awareness grounded in bodily and sensory experience.

This study aims to provide two main contributions. Theoretically, it advances the integration between occupational health psychology and environmental approaches to well-being by conceptualizing surfing as a nature-based resource for coping, recovery, and work–life balance. Practically, it highlights the potential role of extra-work activities, particularly those involving immersion in natural environments, as valuable resources for promoting psychological well-being and preventing psychosocial risks in work settings, while recognizing that such individual resources should complement, rather than replace, organizational responsibility for creating healthy work environments.

## 2. Materials and Methods

An exploratory qualitative approach was adopted to investigate the subjective experiences of workers who practice surfing in their free time, with a particular focus on coping strategies for work-related stress, the development of personal resources, and the dynamics of work–life interface. A qualitative design was chosen to gain in-depth access to participants’ lived experiences, capturing the meanings they attributed to work and the relationship between work and personal life. The reporting of the study was informed by the Consolidated Criteria for Reporting Qualitative Research (COREQ), where applicable, to enhance transparency in reporting the study design, data collection, and analysis procedures [25].

Participants were eligible for inclusion if they were adult workers and had regular experience of surfing as a leisure activity. Participants were contacted through personal and professional networks related to surfing communities and were invited to participate by email or direct message. The invitation included information about the aims of the study, the voluntary nature of participation, and the confidentiality of the data. Interviews were conducted between August 2025 and December 2025. Participants were recruited through purposive sampling to include individuals from diverse professional backgrounds (e.g., healthcare, technical, creative, and managerial roles) who shared a common involvement in surfing. The sample consisted of 14 Italian workers (12 men and 2 women) who regularly engage in surfing during their leisure time. At the time of the interviews, participants were aged between 20 and 40 years. Their professional experience ranged from 2 to 21 years, while their surfing experience ranged from 3 to 30 years. Surfing frequency varied from daily practice to once a month. The sample included freelancers, self-employed workers, and employees with fixed-term or permanent contracts. Participants came from different professional backgrounds, including, for example, engineering, craftsmanship, tourism, corporate work, management, entrepreneurship, healthcare, and IT. The final sample size was evaluated in relation to the exploratory aim of the study, the specificity of the population under investigation, and the depth and relevance of the information provided by participants. Following recent methodological recommendations, saturation was not treated as a fixed numerical threshold, but as a criterion to be defined and reported transparently in relation to the research question, sampling strategy, and analytical process [26]. Recruitment was concluded after 14 interviews because the data provided sufficient informational depth to address the research questions, key patterns recurred across participants’ accounts, and the final interviews did not generate substantially new themes relevant to the aims of the study. Therefore, the sample was considered adequate for the exploratory qualitative aims of the research, while acknowledging that the findings are not intended to be statistically generalizable.

At the beginning of each interview, participants were informed about the aims of the study and provided informed consent, explicitly confirming their voluntary participation. Anonymity and confidentiality were ensured, and no personally identifiable information was collected during the interviews. All interviews were audio-recorded with participants’ consent and subsequently transcribed verbatim. The interviewer adopted a reflective and non-directive stance, encouraging the spontaneous emergence of participants’ narratives while ensuring consistency in the data collection process. The study was conducted in accordance with the Declaration of Helsinki and the General Data Protection Regulation (EU 2016/679) and was approved by the Ethics Committee of the University of Bari.

Data were collected through individual semi-structured interviews conducted via videoconference, with an average duration of 40–60 min. The interview guide was developed through a theory-informed and iterative process, following methodological recommendations for the construction of semi-structured interview guides [27]. First, the authors identified the main conceptual areas derived from the study aims and theoretical framework, namely coping and recovery processes [4,23], work–life interface research [9,24], and literature on nature-based and surf-related experiences [14,15,18,19,22]. These areas were then translated into open-ended questions designed to elicit participants’ narratives about work-related stress, recovery experiences, the perceived role of surfing, and the relationship between work and non-work domains. The guide was discussed and refined by the research team before data collection to ensure clarity, coherence with the research questions, and sufficient openness to allow unexpected themes to emerge. The interview protocol followed a thematic structure organized into five phases, designed to progressively explore participants’ professional and personal experiences:

1. Warm-up phase: general questions about participants’ work, professional role, and job tasks, aimed at establishing rapport and collecting contextual information.
2. Work-related experiences phase: questions focused on daily work activities, sources of work-related stress, and coping strategies adopted.
3. Work–life interface phase: questions exploring the relationship between work and personal life, including perceived balance and potential conflicts between work and personal life.
4. Surfing and extra-work activities phase: questions examining the contribution of surfing to psychological well-being, its role in developing personal resources, and its potential influence on work–life balance.
5. Closing phase: open-ended questions allowing participants to reflect freely and add any additional considerations they found relevant.

The semi-structured interview guide, translated from Italian into English, is provided in Appendix A to enhance transparency and reproducibility.

The interview transcripts were analyzed using thematic analysis following the approach proposed by Braun and Clarke [28]. An inductive and primarily semantic approach was adopted to identify patterns of meaning grounded in participants’ accounts, while remaining informed by the study’s theoretical framework. The analysis followed a recursive process. First, transcripts were read repeatedly to achieve familiarity with the data. Second, initial codes were generated by systematically identifying relevant features across the dataset. Third, codes were organized into preliminary themes reflecting recurring patterns of meaning. These themes were then reviewed and refined through an iterative process, ensuring internal coherence and consistency with the original transcripts. Finally, themes were defined and labeled, and representative excerpts were selected to illustrate each theme. Throughout the analysis, particular attention was paid to maintaining coherence between data and interpretations, adopting a reflexive stance.

The analysis was conducted collaboratively by the authors. Initial coding was first performed on the interview transcripts, and preliminary codes were then discussed among the research team. Disagreements or alternative interpretations were resolved through iterative discussion and comparison with the original transcripts until consensus was reached. Given the qualitative and interpretive nature of the study, analytical rigor was pursued through consensus-based coding, repeated engagement with the data, reflexive discussion among authors, and continuous checking of the coherence between codes, themes, and participants’ narratives. The analysis was conducted manually through systematic reading, coding tables, and iterative comparison across transcripts. This approach was considered appropriate given the manageable size of the corpus and the interpretive aims of the study.

## 3. Results

**Figure 1:**
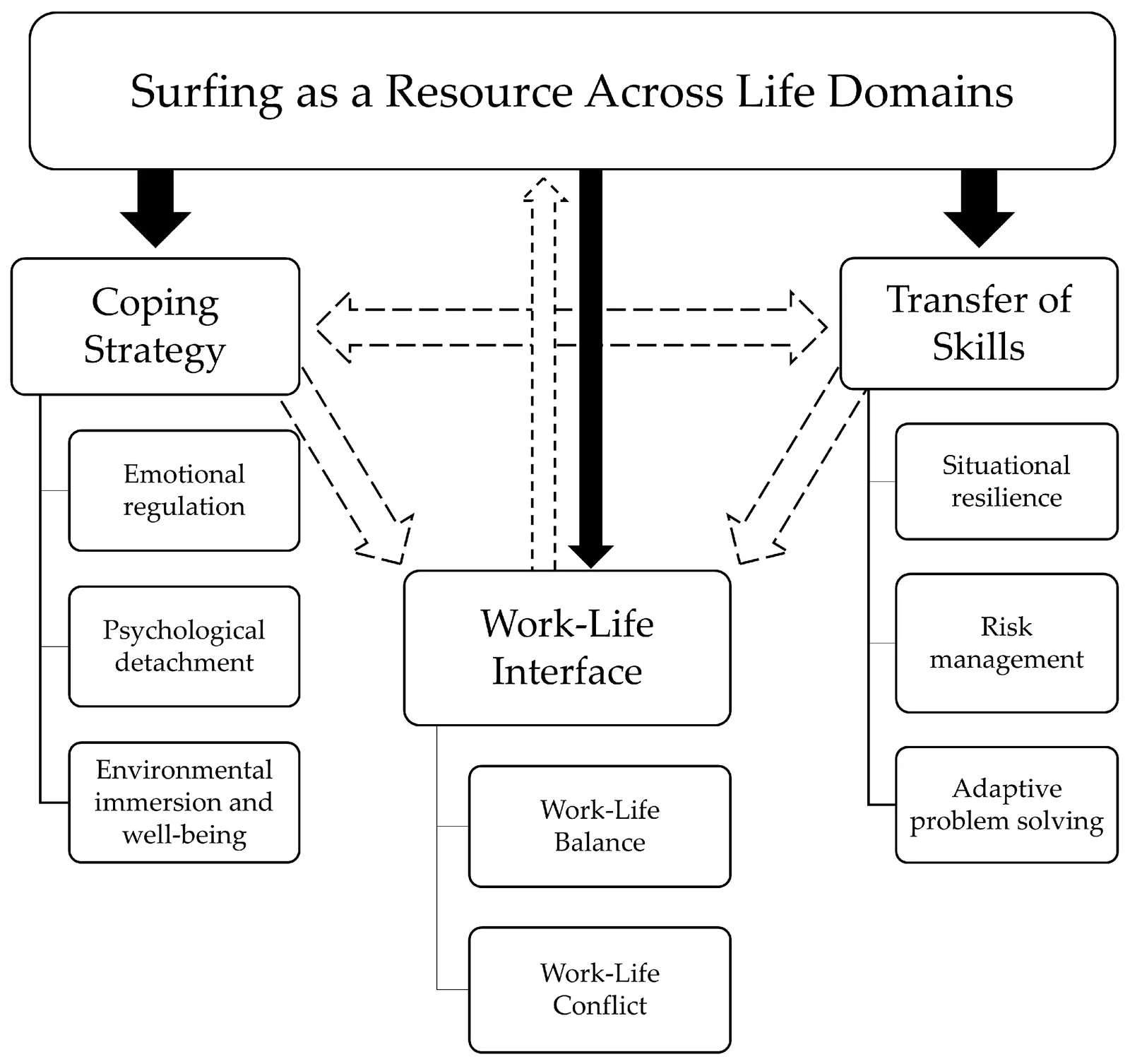
Qualitative explanatory model of surfing as a resource for coping, personal resource development, and work–life dynamics. Note: Solid arrows indicate the main thematic links emerging from participants’ narratives, while dashed arrows represent reciprocal and indirect relationships among coping strategies, transfer of skills, and the work–life interface. The upward dashed arrow indicates that engagement in surfing may also be constrained by work schedules, time demands, and broader life responsibilities. The model should be interpreted as an analytical representation of the qualitative findings, rather than as a causal model.

Three main themes emerged from the analysis: (1) surfing as a coping strategy, (2) the transfer of surfing-to-work skills, and (3) the work–life interface. Together, these themes illustrate how surfing was described as a resource for managing work-related stress, developing transferable skills, and shaping the relationship between work and non-work domains. Figure 1 presents a qualitative explanatory model of the relationships among the main themes and sub-themes emerging from the analysis. The model illustrates how surfing was described as a nature-based leisure activity that supports coping and recovery and fosters personal resource development and transfer. These processes, in turn, contribute to shaping the work–life interface. At the same time, the possibility of engaging in surfing is itself shaped by work schedules, time constraints, and broader life responsibilities. Overall, the figure provides a synthetic overview of the findings and anticipates the themes that are described in detail in the following sections.

### 3.1. Surfing as a Coping Strategy

The first theme that emerged from the analysis highlighted how participants used surfing as a way to cope with perceived workplace stress. A consistent pattern across interviews showed surfing described as an experience capable of restoring emotional balance, reducing the cognitive load associated with work, and fostering a sense of psychological detachment from professional pressures. Within this process, the natural setting of the sea played a central role in enhancing its regenerative effects. The theme was articulated into three sub-dimensions: emotional regulation, psychological detachment, and environmental immersion and well-being.

#### 3.1.1. Emotional Regulation

Participants’ accounts suggested that surfing functioned as a mechanism of emotional regulation, defined as a process through which individuals influence their emotional states, modulating their intensity and duration in line with personal goals and contextual demands [29,30,31]. In the present study, this sub-theme captures the affective dimension of surfing-related recovery, including the down-regulation of negative emotions associated with work-related stress and the regulation of emotions elicited by challenging situations in the water. This differs from psychological detachment, which refers more specifically to cognitive disengagement from work-related thoughts during non-work time. Accordingly, excerpts were interpreted as indicators of emotional regulation when they were primarily concerned with the modulation of affective states, whereas excerpts emphasizing not thinking about work or mentally switching off were considered more closely related to the next sub-theme of psychological detachment. Surfing may act as an emotional regulator because it combines exposure to an aquatic environment (“blue space”), physical activity, and focused attention. Thus, immersion in marine environments can be associated with reduced perceived stress, increased mental recovery, and improved psychosocial well-being, fostering the state of alert calm described as Blue Mind [18,19]. This state may facilitate emotional regulation by reducing cognitive load and promoting present-moment awareness, particularly following a stressful workday. These interpretations were reflected in participants’ narratives:

GD, osteopath (F): “When I get in and out of the water, it’s as if I’ve reset everything, so I no longer have any worries”.

FG, IT professional (M): “When you’re surfing, you can face critical situations: maybe you fall and end up on the reef, or you’re under a wave and can’t get back up. In those cases, you’re forced to control your emotions”.

These statements point to surfing as a tool for emotional regulation. GD’s reference to “resetting” worries suggests a perceived reduction in emotional tension after surfing, while FG explicitly describes the need to control emotions when facing critical situations in the water. Taken together, these accounts show that surfing may support both recovery from affective strain and the regulation of emotions during challenging experiences. In this sense, the sub-theme captures the affective dimension of surfing-related recovery, while the more specifically cognitive suspension of work-related thoughts is addressed in the following sub-theme.

#### 3.1.2. Psychological Detachment

A second sub-dimension concerned the temporary suspension of work-related demands, which can be conceptually linked to psychological detachment from work. Psychological detachment refers to the ability to disengage from work-related thoughts and responsibilities during non-work time [2,4]. It represents a key recovery experience, allowing both physiological activation and cognitive processes triggered by job demands to return to baseline levels, thereby reducing strain and preventing the accumulation of stress and burnout symptoms [1,2,4].

In this context, surfing can be considered as a practical means of facilitating psychological detachment. Engaging in surfing involves clear physical and mental disengagement from the work environment, characterized by the absence of work-related demands and distractions. At the same time, the activity requires sustained attentional focus on the present moment (e.g., reading the sea, positioning oneself, and coordinating movements with the waves), thereby reducing work-related rumination [32,33]. These interpretations were reflected in participants’ narratives:

MM, engineer (M): “It’s an incredible outlet; when I get in the water, I don’t think about anything”.

FR, chef (M): “I can take my mind off things for a moment—it’s an outlet”.

EG, radio professional (M): “In my opinion, it serves to erase everything that might be mentally draining us during the day”.

These accounts suggested that surfing facilitated a temporary suspension of work-related demands by enabling individuals to disengage cognitively from work concerns. The immersive and attention-demanding nature of the activity appeared to limit the mental space available for work-related thoughts, reinforcing a state of focused presence and psychological distance from occupational pressures.

#### 3.1.3. Environmental Immersion and Well-Being

The final sub-dimension concerned environmental immersion and its contribution to well-being. Environmental psychology suggests that natural environments are not neutral contexts but play an active role in promoting psychophysiological recovery. Exposure to natural settings has been associated with reduced physiological arousal, improved mood, and enhanced attentional functioning, compared to more urbanized environments [12,13,34]. In this study, environmental immersion does not refer simply to a general preference for nature. Rather, it captures how participants described the marine environment as an immersive and dynamic setting that required attention, fostered presence, and supported psychological restoration. Within this framework, surfing represents a particularly salient example of the role of the physical environment for well-being, as it combines immersion in a dynamic blue space with physical activity and sustained attentional engagement. These interpretations were reflected in participants’ narratives:

EV, manager (M): “(The physical context) is crucial; I’ve chosen to spend my life in recent years surrounded by nature”.

NP, content creator (M): “Surfing can be considered a spiritual experience. However we move our bodies, we are in total presence, connection, listening, and even understanding of the element of water: seeing how the waves move, how the tide is moving, the wind, the direction of the swell; a deep understanding of the place”.

FD, chef (M): “A beautiful place to surf makes all the difference (for my well-being)”.

These accounts highlight the central role attributed to the natural environment in shaping the surfing experience and its perceived benefits. EV’s reference to the importance of living “surrounded by nature” indicates that the physical context was experienced as a meaningful condition for well-being. NP’s account further specifies this mechanism by referring to concrete environmental cues, such as waves, tides, wind, and swell direction, which require attention and foster presence. Similarly, FD explicitly connects the quality of the surfing place with perceived well-being. The environment is not described merely in aesthetic terms, but as a dynamic and interactive context that fosters connection, presence, and psychological restoration.

### 3.2. Transfer of Surfing-to-Work Skills

The second theme concerned the transfer of skills developed through surfing to the work domain. Participants’ narratives revealed that surfing was not experienced merely as a recreational activity, but as a context that fostered the development of personal resources relevant to the workplace, such as frustration tolerance, management of uncertainty, self-regulation, and decision making. These skills were described as being transferred both implicitly and consciously to professional contexts.

Within this perspective, surfing was often portrayed as a “training ground” in which individuals learned to navigate dynamic and unpredictable environments, drawing parallels between ocean conditions and organizational demands. The theme was articulated into three sub-dimensions, including situational resilience, risk management, and adaptive problem solving.

#### 3.2.1. Situational Resilience

Situational resilience refers to the context-specific capacity to maintain or regain effective functioning when facing adversity, rather than a stable individual trait [35,36,37]. This perspective emphasizes that resilience may vary across life domains and situations, reflecting the interaction between individual resources and contextual demands. Within this framework, surfing can be interpreted as a context that fosters the development of situational resilience. The activity repeatedly exposes individuals to unpredictable and challenging conditions, such as changing waves and environmental variability. These repeated cycles of challenge may contribute to the development of more flexible resilient responses and increased confidence in dealing with demanding situations. Such experiences may be transferred to the work domain, where similar capacities are required to manage workload peaks, role demands, and uncertainty. These interpretations are reflected in participants’ narratives:

ML, manager (F): “(Surfing) develops your patience, self-esteem, and emotional resilience”.

FR, entrepreneur (M): “If companies made surfing (or any other sport with these principles) more accessible, employees would certainly be more motivated to practice it. And in doing so, they would automatically learn these values of resilience, of stepping out of their comfort zone, and of not getting angry”.

NP, radio professional (M): “From surfing, I’ve learned to accept things as they are, to listen to myself, not to rush”.

These accounts suggested that surfing supported the development of situational resilience by encouraging acceptance of uncontrollable conditions, patience, and persistence. Participants explicitly linked experiences in the water to how they approached challenges in other life domains, including work. The ability to tolerate uncertainty and recover from setbacks emerged as a transferable resource, contributing to how individuals managed demanding and unpredictable situations in their professional lives.

#### 3.2.2. Risk Management

Risk management refers to the process through which individuals perceive, assess, and regulate their exposure to potentially risky situations, balancing perceived costs and benefits [38,39]. From this perspective, risk is not eliminated but managed through the development of specific strategies, involving anticipation, situational awareness, and decision making aimed at maintaining risk within acceptable limits [40,41]. Research on high-risk sports suggested that people do not seek risk indiscriminately but rather engage in deliberate preparation and evaluation processes to minimize negative outcomes while preserving an appropriate level of challenge [38,42].

Within this framework, surfing can be interpreted as a context that fosters the development of risk management skills. The activity requires continuous evaluation of contextual conditions (e.g., waves, tides, wind), as well as the alignment between these conditions and one’s own capabilities. Such assessments translate into ongoing decisions about whether and how to engage with the environment, promoting the ability to distinguish between controllable and uncontrollable elements. This process appears to support the development of a more reflective and calibrated approach to risk, which may be transferable to work-related situations involving decision making under constraints. These interpretations were reflected in participants’ narratives:

FG, IT professional (M): “We need to distinguish between types of difficulty, because there are difficulties you can view as challenges, and difficulties that remain just that because they don’t depend on you. The sea doesn’t depend on you; we don’t control it, we don’t control the waves, but the paddle, the take-off, staying on the board: those are things we can improve. When, for example, you go into the water with rough seas, with particularly adverse conditions, you feel powerless, and you can’t do anything other than put into practice what you’ve learned”.

MZ, artisan (M): “Another issue, on the other hand, is that in conditions that are maybe big, demanding, where I don’t feel comfortable, well, I’m in the water, but instead of staying out for two hours, I stay for one, and I’m happy to have survived” [ironic].

These accounts suggested that surfing fostered an awareness of both the limits of control and the need to calibrate one’s exposure to risk. Participants described a process of learning to differentiate between situations that could be actively managed and situations that required acceptance, withdrawal, or adjustment. This orientation toward calibrated risk appeared to extend beyond the surfing context, informing how individuals approached uncertainty and constraints in other life domains.

#### 3.2.3. Adaptive Problem Solving

Adaptive problem solving refers to the set of cognitive and behavioral processes through which individuals interpret situational cues, evaluate possible courses of action, select appropriate responses, and adjust their behavior as conditions change [43,44]. In this study, adaptive problem solving was distinguished from risk management because it does not primarily concern the calibration of exposure to potentially hazardous conditions. Rather, it refers to the broader process of reading a situation, identifying possible responses, choosing the appropriate timing, and revising one’s actions in light of changing circumstances.

Within this framework, surfing can be interpreted as a context that fosters the development of problem-solving skills. Surfers must interpret multiple cues (e.g., wave patterns, positioning, timing) and adjust their actions accordingly, often in situations characterized by complexity and limited control. This ongoing process of evaluation, choice, and adaptation reflects core components of adaptive problem solving and may support the development of transferable cognitive and behavioral strategies applicable to work-related contexts. These interpretations were reflected in participants’ narratives:

FD, chef (M): “Surfing teaches us to choose carefully, to figure out if that’s the right wave or not. So, knowing how to wait, knowing how to choose well, and not being impulsive. I learned that on the board. A situation might arise that needs to be handled calmly rather than tackled head-on impulsively, so it might be better to pause for a moment, think it through, and see what’s behind it, to turn that situation to my advantage”.

AV, tour operator (M): “(From surfing) I take with me taking things more lightly, that is, expecting less from myself. What surfing makes you realize is that you’re mortal, you’re not superior to anyone, you’re exactly the same as everyone else. I’d actually already understood this, but surfing brought it home to me. And then one thing it’s teaching me more and more is patience, the ability to wait for the right moment”.

These accounts suggested that surfing supported adaptive problem-solving processes since participants described learning to pause, assess situations, and avoid impulsive reactions, emphasizing the importance of aligning actions with changing conditions. In contrast to risk management, which concerned the calibration of exposure to uncertain or potentially demanding conditions, adaptive problem solving captured how participants translated situational reading into concrete choices and actions. Such experiences appeared to foster a more reflective and flexible approach to problem-solving, which could extend to how individuals manage complex situations at work.

### 3.3. Surfing and the Work–Life Interface

The third theme concerned the role of surfing within the work–life interface. Participants’ accounts revealed a dual and dynamic relationship between work and non-work domains. On the one hand, work demands could constrain the time, energy, and resources available for surfing; on the other hand, surfing could emerge as a source of personal resources that enhance psychological well-being and contribute positively to the work domain.

This bidirectional dynamic reflected both potential tensions and positive spillover processes between life domains, highlighting how an extra-work activity such as surfing could simultaneously generate constraints and resources. In particular, surfing was described as influencing both experiences outside of work and attitudes and functioning within the work context. The theme was articulated into two sub-dimensions: work–life balance and work–life conflict.

#### 3.3.1. Work–Life Balance

Work–life balance refers to the perceived equilibrium between work and non-work roles, in which individuals are able to effectively manage the demands of both domains without experiencing excessive strain or conflict [24]. This balance is not defined by an equal distribution of time, but by a subjective sense of compatibility between professional responsibilities and personal activities.

Within this framework, surfing could function as an activity that individuals integrate into their daily routines, often through deliberate organization of work schedules and personal time. Participants described adopting strategies aimed at preserving space for leisure, suggesting a proactive management of boundaries between work and non-work domains. These interpretations were reflected in participants’ narratives:

FD, chef (M): “Once I’m done, if there’s a chance to do some sport, in my case surfing, I grab my board and go, conditions permitting. I make sure to organize my work so that I’ll be free during that time, though I always prioritize my work first and foremost”.

AV, tour operator (M): “I have an excellent balance, a classic work-life balance, so I really enjoy my free time, because I know that if I have free time, it means I’ve done everything I need to do, since I’ve organized it properly”.

EG, radio professional (M): “Fortunately, these two spheres (work and personal) coexist. On weekends and in the evenings, once everything is done, there’s more time for what I enjoy doing”.

These accounts suggested that work–life balance was achieved through the active organization of time and priorities, rather than through a fixed distribution of resources between domains. Participants emphasized the importance of structuring work commitments in a way that allowed engagement in personally meaningful activities, such as surfing.

#### 3.3.2. Work–Life Conflict

Work–life conflict refers to a form of inter-role conflict in which the demands of work and non-work domains are mutually incompatible, such that participation in one role makes engagement in the other more difficult [9]. This conflict may take different forms, including time-based conflict (i.e., when time devoted to one domain reduces availability for the other) and strain-based conflict (i.e., when stress or fatigue experienced in one domain interferes with functioning in the other). Research on demanding leisure activities suggested that conflict may emerge when time and energy investments are not aligned with broader role demands [24].

Within this framework, surfing may both alleviate and contribute to work–life conflict. While it can support recovery and reduce strain, it may also generate time-based conflict when it requires specific and often inflexible time windows linked to environmental conditions (e.g., swell, tides, wind). The need to adapt to these conditions may clash with work schedules and other responsibilities, creating tensions between competing demands. These interpretations were reflected in participants’ narratives:

EG, radio professional (M): “(My private and work life are in conflict) when you’re doing so many things, there’s automatically less space for ‘extra’ things”.

GM, photographer (M): “Personally, I’d like not to have to sacrifice work for surfing”.

ML, manager (F): “Right now, (work and personal life) overlap too much. The more work there is, the more I realize how much I’m currently trying to draw small, albeit faint, boundaries around my personal life, which involves surfing, which involves people”.

These accounts suggested that surfing could contribute to work–life conflict when time constraints and role demands became difficult to reconcile. Participants described the need to negotiate boundaries between work and personal activities, highlighting how competing priorities could limit the availability of time and energy. At the same time, the narratives reflected an awareness of these tensions and an effort to manage them through boundary-setting and prioritization.

## 4. Discussion

This study aimed to explore how adult workers who regularly practice surfing experience this activity as a resource for coping with work-related stress and shaping work–life dynamics, drawing on data collected through semi-structured interviews. The thematic analysis revealed three main themes: surfing as a coping strategy, the transfer of skills between surfing and work, and the role of surfing in the work–life interface. Although analytically distinct, these themes were closely intertwined in participants’ narratives. The explanatory model presented in Figure 1 helps clarify this interconnection. Surfing emerged as a nature-based leisure activity that combines environmental immersion, physical engagement, sustained attentional focus, and exposure to changing conditions. These features appear to support coping and recovery processes, particularly through emotional regulation and psychological detachment. At the same time, repeated engagement with the uncertainty and demands of the sea may foster personal resources, such as resilience, risk management, and adaptive problem solving, which participants perceived as transferable to the work domain. The work–life interface is therefore not only an outcome shaped by surfing-related recovery and resource development, but also a contextual condition that influences whether and how individuals can engage in surfing, depending on work schedules, time constraints, and broader life responsibilities. Overall, the findings suggested that, for these workers, surfing was not merely a recreational activity but a psychosocial resource through which work-related stress was managed, personal resources were developed, and the boundaries between professional and private life were negotiated.

Regarding the first theme, surfing as a coping strategy, the findings indicated that surfing was experienced as an effective means of recovery from work-related stress and demands. Participants described surfing as an activity that “erases” mental load or allows a “reset” of worries, suggesting a form of coping primarily oriented toward the regulation of internal states. At the same time, the strong physical engagement and attentional focus required by the activity facilitated a temporary suspension of work-related thoughts, consistent with the concept of psychological detachment from work. Surfing thus emerged as a powerful recovery experience, characterized by both physical and mental disengagement from the work context. The natural setting, and particularly the marine environment, further amplified these effects, as participants consistently emphasized the importance of being immersed in nature. Importantly, these recovery processes also appear to create psychological conditions through which participants could re-enter work with greater emotional balance and perceived self-regulation. This is aligned with research in environmental psychology, which highlighted the restorative properties of natural environments in reducing stress and replenishing attentional resources [12,13,33,34].

The second theme, concerning the transfer of surfing-to-work skills, highlighted how surfing could function as a context for the development of personal resources that extend beyond the activity itself. Rather than being perceived solely as an outlet, surfing was described as a setting in which individuals repeatedly engaged with uncertainty, failure, and environmental constraints, thereby fostering capabilities such as patience and tolerance of frustration. These experiences appeared to support the development of situational resilience, which participants recognized as relevant in managing work-related challenges. Additionally, risk management represented another area of transfer. Participants described learning to distinguish between controllable and uncontrollable elements, to assess conditions before acting, and to calibrate their level of exposure to risk. This reflected a shift toward a more reflective and less impulsive approach to decision making, which was also applied in professional contexts. Similarly, surfing was experienced as a continuous process of problem solving, requiring the interpretation of complex and dynamic conditions and the selection of appropriate actions under uncertainty. These resources were closely connected to the coping and recovery processes described above, as the ability to regulate emotions and remain focused in challenging conditions appeared to support participants’ perceptions of resilience, risk management, and adaptive problem solving. These findings suggested that surfing may contribute to the development of cognitive and behavioral strategies that were transferable to work-related situations, particularly those characterized by complexity and unpredictability.

Finally, the third theme concerned the relationship between surfing and the work–life interface, highlighting the ambivalent role of this activity in the management of work and non-work domains. On the one hand, surfing was described as a key resource for maintaining balance, as participants actively organized their work to create space for leisure and experience their free time as more meaningful and restorative. On the other hand, elements of work–life conflict also emerged, particularly when surfing required adherence to specific environmental conditions that were not easily compatible with work schedules. In such cases, the need to “chase the waves” could conflict with professional and personal responsibilities, generating time-based tensions. This ambivalence is central to understanding surfing across life domains because the same activity that supports recovery and resource development may also require time, flexibility, and boundary management, thus creating potential tensions between work and non-work domains.

Overall, the findings suggested that surfing served a dual function: it acted as a coping and recovery resource in response to work demands, and as a context for the development of personal resources that could be transferred across life domains. At the same time, its integration into everyday life was not without challenges, as it could also generate tensions when demands from different domains were difficult to reconcile.

### 4.1. Theoretical Implications

From a theoretical perspective, the results of this study offer four main contributions. First, the findings contribute to integrating perspectives that are often treated separately in the literature, namely coping processes [4,23], work–life interface research [9], and studies on nature-based and blue-space activities [18,45]. By showing how surfing is used both as a strategy for emotional regulation and psychological detachment, and as a context for the development of personal resources, this study builds a conceptual bridge between occupational health psychology and research on nature-based experiences. This highlights how processes typically examined within work contexts (e.g., coping and resource development) can also be enacted within nature-based extra-work environments. More specifically, the study extends coping theory by showing that coping with work-related stress may occur not only through cognitive or behavioral strategies directed at work demands, but also through embodied and environmentally embedded experiences outside work. In this sense, surfing emerged as a coping context in which physical engagement, contact with the sea, attentional focus, and emotional regulation were closely intertwined. This suggests that nature-based leisure activities may support coping not simply because they provide time away from work, but because they create specific experiential conditions that facilitate recovery and self-regulation.

Second, the findings support a process-oriented and situated understanding of resilience, risk management, and problem solving. Rather than emerging as stable individual traits, these capacities appear to be dynamically developed through repeated engagement with extra-work contexts characterized by unpredictable and physically demanding environments, such as the sea. These capacities can then be transferred across domains, enhancing workplace functioning through enhanced adaptability and decision-making processes [35,36,37]. From this perspective, surfing can be interpreted as an embodied context in which individuals learn to tolerate uncertainty, regulate emotions, and manage controlled risk, thereby contributing to the development of flexible behavioral and cognitive responses. This represents a conceptual contribution to research on personal resources by showing how resources relevant to work may be developed in non-work contexts and later mobilized in professional situations. In this respect, the findings extend resource-based perspectives on the work–life interface, including the Work–Home Resources Model [11], by illustrating how extra-work experiences may generate resources that move across domains. Surfing was therefore not only experienced as a recovery activity, but also as a setting for learning how to tolerate uncertainty, regulate emotions, assess risk, and solve problems adaptively.

Third, the study refines the understanding of the work–life interface by moving beyond a simple dichotomy between work and leisure. Surfing emerges as an activity that can simultaneously support balance and generate conflict. On the one hand, it facilitates recovery and psychological detachment, contributing to the maintenance of well-being [2,4]; on the other, it may generate time-based tensions with work and personal responsibilities [9]. These findings suggest that extra-work activities should not be considered uniformly beneficial, but rather understood in relation to their temporal demands, degree of personal significance, and compatibility with individuals’ role systems. This contribution is relevant because it shows that the same leisure activity may function both as a resource and as a demand within the work–life interface. Thus, surfing does not simply enrich or interfere with work and personal life in a linear way; rather, its effects depend on how individuals negotiate boundaries, schedules, responsibilities, and access to environmental conditions.

Fourth, the study advances previous qualitative research on surfing and occupational well-being by proposing an explanatory model of the relationships among the identified themes. The explanatory model suggests that coping and recovery processes, transfer of skills, and work–life dynamics are not isolated outcomes, but interrelated dimensions of the same experience. Surfing may support recovery and emotional regulation, these processes may foster the development of personal resources, and such resources may be transferred to work while also shaping the way individuals manage boundaries between work and non-work domains. At the same time, work–life conditions may constrain access to surfing, showing that the relationship between leisure and occupational well-being is reciprocal rather than one-directional. This represents a theoretical contribution because it moves beyond a descriptive account of surfing’s benefits and conceptualizes surfing as a dynamic process operating across life domains. In doing so, the model provides a more integrated understanding of how nature-based leisure activities may contribute to occupational well-being through interconnected recovery, resource development, and work–life processes.

### 4.2. Practical Implications

The findings of this study suggest practical implications at the organizational and individual levels. For organizations and HR departments, the results highlight the importance of complementing in-house well-being initiatives with a broader understanding of how employees recover outside the workplace. Leisure activities that involve immersion in natural environments and sustained attentional focus, such as surfing, should be recognized as meaningful resources for coping and recovery. In this regard, organizational practices such as flexible scheduling and remote work arrangements may support the sustainable integration of such activities into daily routines, thereby reducing the risk of time-based conflict. At the same time, these findings should not be interpreted as shifting responsibility for managing work-related stress onto individual workers. Activities such as surfing may support recovery and personal resource development, but they cannot replace organizational responsibility for preventing psychosocial risks, reducing excessive job demands, and creating healthy work environments. From this perspective, individual recovery practices and organizational interventions should be understood as complementary rather than substitutive.

Moreover, managerial training programs may benefit from explicitly addressing concepts such as emotional regulation and recovery, including concrete examples of how employees manage stress through extra-work experiences. This may help managers better recognize and support employees’ recovery processes, rather than overlooking these activities as unrelated to work performance. At the same time, greater attention to employees’ extra-work experiences may also support the recognition and development of transferable competencies, such as resilience, decision making under uncertainty, and problem solving, which can contribute to more effective functioning in the workplace.

For individuals, the findings highlight the active role that workers may play in recognizing and cultivating meaningful recovery experiences outside work, without implying that responsibility for managing occupational stress lies primarily with individuals. Participants report actively using extra-work activities to regulate stress, create psychological distance from work, and maintain a sense of balance across life domains. This suggests the importance of developing greater awareness of how extra-work activities function as personal resources, as well as the ability to integrate them into daily routines. In addition, recognizing how experiences outside work contribute to the development of competencies (e.g., managing uncertainty and regulating emotions) may support a more effective use of these resources in work-related situations.

### 4.3. Limitations and Future Directions

This study presents several limitations that should be considered when interpreting the findings. First, the small sample size and its composition, characterized by a predominance of male participants and by the specificity of individuals who regularly engage in surfing, limit the generalizability of the results. Moreover, all participants were Italian workers. Although the study did not aim to examine national culture as a central analytical dimension, the findings should be interpreted in light of this context. Work–life boundaries, access to coastal environments, and the cultural meanings associated with surfing may differ across countries and occupational settings, limiting the transferability of the findings. Future research should involve larger and more diverse samples, including a more balanced gender distribution, a wider range of occupational sectors, different types of leisure activities, and different cultural contexts.

Second, the qualitative design, based on self-reported experiences, may be subject to social desirability bias and to a potential emphasis on the positive aspects of surfing. Future studies could address this limitation by adopting mixed-method approaches, integrating qualitative data with quantitative measures, as well as incorporating external or multi-source assessments. A related limitation concerns potential self-selection bias. Participants were already regular surfers and may therefore have held favorable attitudes toward surfing and its perceived benefits. As a consequence, positive experiences of recovery, well-being, and resource development may be overrepresented in the data. Future studies should compare surfing with other meaningful leisure activities, including non-nature-based activities and other forms of physical activity, to better understand which mechanisms are specific to surfing and which may characterize leisure experiences more broadly.

A further limitation concerns the interpretive nature of the analysis, which may be influenced by the researchers’ perspectives. Although efforts were made to ensure coherence and transparency in the analytical process, future research could further strengthen the robustness of findings through additional validation procedures.

Finally, the findings should not be interpreted as evidence of the effectiveness of surfing as an intervention for occupational stress or public health. The study provides an exploratory qualitative account of workers’ lived experiences and perceived meanings, rather than causal evidence. Future research could adopt intervention designs to examine whether and under what conditions nature-based leisure activities contribute to measurable changes in psychological well-being and work-related outcomes.

Despite these limitations, the study provides a coherent account of how surfing may function as a resource for coping with work-related stress, supporting recovery processes and shaping the work–life interface. These findings offer a basis for future research aimed at further examining the role of nature-based activities in occupational well-being, as well as for the development of interventions that recognize and integrate extra-work experiences as part of broader well-being strategies.

## 5. Conclusions

This study provides a qualitative account of how surfing, as a nature-based leisure activity, may function as a resource in the context of occupational well-being. The findings show that participants actively use surfing as a coping strategy to regulate stress and facilitate psychological detachment from work, while also drawing on competencies developed in this context to support functioning in the workplace. At the same time, surfing emerges as a relevant component of the work–life interface, contributing to the maintenance of balance but also, under certain conditions, generating tensions related to time constraints and role demands.

Taken together, these results suggest that extra-work activities should not be considered peripheral to work functioning, but rather as contexts in which individuals actively develop and mobilize personal resources. In particular, activities involving immersion in natural environments and high levels of personal engagement may offer unique opportunities for recovery, self-regulation, and adaptive functioning across domains.

Overall, the study contributes to a growing body of research emphasizing the role of nature-based experiences in supporting psychological well-being [18,19,32], highlighting the need to more fully integrate extra-work contexts into models of occupational health. At the same time, the exploratory nature of the study calls for caution in translating these findings into generalized recommendations. Nature-based leisure activities may represent valuable complementary resources for well-being, but they should be considered alongside organizational strategies aimed at preventing psychosocial risks and promoting healthier work environments.

## Data Availability

The data presented in this study are not publicly available due to privacy and ethical restrictions, as they contain information that could compromise the confidentiality of research participants.

## Appendix A. Semi-Structured Interview Guide

Note that the interviews were conducted in Italian. The following guide is an English translation of the semi-structured interview guide used in the study. The guide was used flexibly, allowing the interviewer to adapt the order of questions and avoid repetition when topics had already been addressed by participants.

1. Warm-up and professional background
  - Could you briefly describe your work?
  - What is your current role and what are your main responsibilities?
  - Could you describe a typical working day?
  - How is your day usually organized between work and other activities?
  - How long have you been working in your current sector?
2. Work-related experiences and stress management
  - What are the main sources of stress or pressure that you encounter in your work?
  - Would you describe your work as stressful? If so, what consequences does work-related stress have for you?
  - How do you usually deal with difficulties or stressful situations at work?
  - What do you usually do when you feel stressed?
  - Do you feel that your organization or work context supports your well-being?
3. Work–life interface
  - How do you manage work and non-work commitments in your daily life?
  - Do you feel that work and private life ever come into conflict?
  - Do you feel that work and private life can also positively influence each other?
4. Extra-work activities: focus on surfing
  - How long have you been practicing surfing? How often do you usually practice surfing?
  - How did you start practicing surfing, and what led you to this activity?
  - What does surfing represent for you?
  - Have you ever perceived improvements in your mood or mental health after surfing?
  - Can you describe whether and how surfing helps you deal with work-related stress?
  - Do you perceive any connections between the way you face waves and the way you face challenges in life or at work?
  - Are there any skills or abilities that you have developed through surfing and transferred to your work? If so, how useful have they been?
  - Do you think surfing has changed your way of thinking or the way you approach difficulties?
5. Closing questions
  - In your opinion, what role could surfing have in promoting well-being in work contexts?
  - Is there anything important that we have not discussed and that you consider relevant to your well-being, surfing, or working life?
  - Would you like to add anything else?
